# The transcriptomic insight into the differential susceptibility of African Swine Fever in inbred pigs

**DOI:** 10.1038/s41598-024-56569-2

**Published:** 2024-03-11

**Authors:** Mohammad Hossein Banabazi, Graham Freimanis, Lynnette C. Goatley, Christopher L. Netherton, Dirk-Jan de Koning

**Affiliations:** 1 Department of Animal Biosciences, Swedish University of Agricultural Sciences, Box 7023, 750 07 Uppsala, Sweden; 2 The Pirbright Institute, Ash Road, Woking, Surrey GU24 0NF UK

**Keywords:** Genetics, Computational biology and bioinformatics

## Abstract

African swine fever (ASF) is a global threat to animal health and food security. ASF is typically controlled by strict biosecurity, rapid diagnosis, and culling of affected herds. Much progress has been made in developing modified live virus vaccines against ASF. There is host variation in response to ASF infection in the field and under controlled conditions. To better understand the dynamics underlying this host differential morbidity, whole transcriptome profiling was carried out in twelve immunized and five sham immunized pigs. Seventeen MHC homozygous inbred Large white Babraham pigs were sampled at three time points before and after the challenge. The changes in the transcriptome profiles of infected animals were surveyed over time. In addition, the immunization effect on the host response was studied as well among the contrasts of all protection subgroups. The results showed two promising candidate genes to distinguish between recovered and non-recovered pigs after infection with a virulent African swine fever virus (ASFV) pre-infection: HTRA3 and GFPT2 (padj < 0.05). Variant calling on the transcriptome assemblies showed a two-base pair insertion into the ACOX3 gene closely located to HTRA3 that may regulate its expression as a putative genomic variant for ASF. Several significant DGEs, enriched gene ontology (GO) terms, and KEGG pathways at 1 day and 7 days post-infection, compared to the pre-infection, indicate a significant inflammation response immediately after ASF infection. The presence of the virus was confirmed by the mapping of RNA-Seq reads on two whole viral genome sequences. This was concordant with a higher virus load in the non-recovered animals 7 days post-infection. There was no transcriptome signature on the immunization at pre-infection and 1 day post-infection. More samples and data from additional clinical trials may support these findings.

## Introduction

African swine fever virus (ASFV) is rapidly spread between animals and causes a lethal hemorrhagic fever with high mortality rates in domestic and wild pigs^1^. It is responsible for massive losses in pig populations with drastic economic consequences. This disease is endemic in Africa, newly emerging in the EU, Asia and the Caribbean, and a global threat^2,3^. According to the 35th situation report of World Organization for Animal Health^4^ published in June 2023, since January 2021, and as of 27 April 2023, ASF has been reported in 47 countries in five different world regions, globally affecting more than 924,000 pigs and more than 26,000 wild boars with more than 1,283,000 animal losses (deaths plus animals killed and disposed of, not including the animals culled in areas around the outbreak for controlling the disease)^4^. Case fatality rates approach 100% after infection with virulent isolates. However, infection with attenuated viruses can show a much less severe disease course, and in many cases, recovered animals are fully protected from subsequent challenges with related virulent viruses^5^.

The dynamics of the differential recovery to a carefully controlled artificial ASFV infection can be studied through transcriptome profiling. The results suggest a way to breed pigs that are more genetically resilient to ASF. The clinical records may support differentially expressed genes (DEGs) conclusions.

The transcriptome of the various genotypes of ASF virus isolate^6–8^, its vector-borne tick^9^, and infected swine macrophages as the host–pathogen interaction^10–12^ have already been studied. A few studies have surveyed the response to the infection in the host^13–16^. None of the latter studies have designed an infection challenge to study the transcriptome signatures of the immunization.

## Materials and methods

### Ethics statement

This present study is reported in accordance with ARRIVE guidelines (https://arriveguidelines.org). The animal experiment has been described previously^5^ and was carried out under the Home Office Animals (Scientific Procedures) Act (1986) (ASPA) and were approved by the Animal Welfare and Ethical Review Board (AWERB) of the Pirbright Institute. All animal housing, challenging, and sampling procedures were conducted by trained and competent Personal License holders who were under the auspices of Project License PPL70/8852.

### Animals and infection challenge

ASFV strains were cultured and titrated using endpoint dilution on bone marrow-derived macrophages as described previously^17^. Seven female and 10 male fifteen weeks old MHC homozygous inbred Large white Babraham pigs^18^ were bred at Animal Plant Health Agency, APHA Weybridge, UK. Twelve pigs were randomly immunized with a low virulent ASFV isolate (OUR T1988/3), and the remaining five were inoculated with phosphate buffer saline (PBS) as the sham vaccine treatment. All animals were challenged with the virulent ASFV isolate (OUR T1988/1) 18 days after the immunization. The dose of OUR T1988/3 for the immunization and OUR T1988/1 for the challenge on day 18 was 10,000 units. Both inoculations were via the intramuscular route and the pigs were studied for cellular and humoral responses after immunization^5^. Scoring of clinical signs and macroscopic lesions assessed at post-mortem were as described previously^19,20^. Virus in the blood and tissues was titrated using quantitative PCR^21^. The schematic experimental design is shown in Supplementary Fig. 1.

### Blood sampling

The biobank blood samples from the study of Goatley et al. (2022) were used in the present study. As it has been described previously^5^, peripheral blood mononuclear cells (PBMC) were prepared from heparinized blood using Histopaque® (Sigma-Aldrich, Merck Life Science UK Limited) and frozen in foetal calf serum supplemented with 10% DMSO. Blood samples were collected pre challenge with OUR T1988/1 (day-1), 1 day post-infection and either when pigs reached their humane endpoint or 7 days post-infection. This latter was included under “7 days post-infection (dpi)” treatment in the data analysis.

### RNA-sequencing

Frozen PBMCs were defrosted into RPMI media supplemented with 10% foetal calf serum. Total RNA was extracted from one million cells using a MagMAX mir Vana Total RNA extraction kit on a MagMAX™ Express-96 Deep Well Magnetic Particle Processor. Prior to sequencing library preparation, quality control was performed by RNA size analysis, on a 4200 Tapestation using RNA ScreenTape (Agilent Technologies, Santa Clara, USA). All samples exhibited RIN values ranging between 7.4 and 9.1 and were quantified using a Qubit RNA BR (ThermoFisher Scientific, Waltham, MA). Sequencing library preparation was performed using the Illumina Stranded mRNA Prep kit (Illumina, San Diego, USA) according to the manufacturer's instructions and automated using a Hamilton NGStar (Hamilton, Bonaduz, SW). Sequencing library quality was assessed using Tapestation 4200 (Agilent Technologies, Santa Clara, USA) and Qubit (ThermoFisher Scientific, Waltham, MA) before being diluted to 5 µM. Libraries were randomly split into nine pools of 8–10 samples. Pooled libraries were quantified using the NEBNext Illumina library quantitation kit (NEB, Ipswich, USA) and Qubit (ThermoFisher Scientific, Waltham, MA) and adjusted to 5 µM prior to spiking with 1% PhiX (Illumina, San Diego, USA). Pooled libraries were sequenced on two 2 × 75 cycle paired-end sequencing runs on a NextSeq 550 System (Illumina, San Diego, USA).

### Data quality control

Two separate RNA libraries were sequenced for each sample that were merged later during analysis. In total, 43 whole RNA-Seq data were qualified by FastQC v0.11.8. The 75-bp paired reads were then trimmed in two steps by Trimmomatics 0.39^22^ and bbduk.sh script available in BBMAP suite v38.94^23^, respectively.

### Transcriptome assembly

The schematic computational workflow is shown in Supplementary Fig. 1. The filtered reads were mapped on the Swine reference genome (Sus_scrofa.Sscrofa11.1, Ensemble release 106) using HISAT2 aligner^24^. In addition, host mRNA reads were mapped against two whole genomes of the ASFV isolates used in this study (Supplementary Materials S1 & Materials S2), including the Portugal_1988-OURT88-3 sequence^25^ (Accession no: NC_044957) is the low virulent strain that was used for the immunizations and the Spain_1975_E75 sequence^26^ (Accession no: NC_044958) is probably very similar to the virulent OUR T1988/1 strain used for the challenge.

### Feature counting

All exon features were extracted from swine gene transfer format (GTF) annotation (Sus_scrofa.Sscrofa11.1, Ensemble release 106) and counted on the individual assembled transcriptomes by featureCounts v2.0.1^27^. Using the DESeq2 v1.32.0 package^28^ under R v. 4.1.3^29^, the count of reads was normalized per sample. The samples were then grouped by the Principle Component Analysis (PCA) under the following model and ordinated on a heatmap.

$${\text{design}} = \sim {\text{group}}$$

That group is the combination of ”Condition_Day".

### Differential Gene Expression (DGE)

Differential Gene Expression (DGE) analysis was performed using the DESeq2 v1.32.0 package^28^ under R v. 4.1.3^29^. All contrasts were covered in the following model.

$${\text{design}} = \sim {\text{condition}}$$

The condition is the host response to infection challenge (recovered vs. non-recovered) or immunization status (vaccine vs. sham). Non-recovered animals were totally twelve pigs, including five pigs of sham (control) group and seven of immunized ones.

The differential gene expression between protected and sham animals was contrasted at each time point with the recovered and sham pigs as the reference group, respectively. The contrasts among the subgroups of immunization-response combinations, including vaccinated-recovered, vaccinated-non-recovered, and sham at each time point, were investigated for more resolution.

In order to address the host response to ASF infection, the comparisons over time (time-series) after the infection challenge were made on the following model by pair in each post-infection time against pre-infection as the base using Likelihood-Ratio Test (LRT) and Day as a reduced factor.

$${\text{design}} = \sim {\text{condition}} + {\text{day}} + {\text{condition:day}}$$

That day is the sampling time in three levels (pre-infection, 1 day, and 7 days post-infection), and condition:day is the interaction between condition and time.

### Gene ontology and pathway enrichment analysis

Gene ontology (GO) and pathway enrichment analyses were done on the Panther database^30^ and interpreted under statistical threshold Bonferroni adjusted P-value equal to and less than 0.05.

### Variant calling

Variant analysis was done on the transcriptome assemblies by bcftools mpileup^31^. In total, two vcf files were individually produced for all recovered and all non-recovered pigs. The variants were visualized and tracked by the Integrative Genomics Viewer (IGV) v.2.14.1^32^ in areas neighboring the significantly differentially expressed genes.

## Results

### Animal trials

The daily clinical signs, body temperature, virus titer (viremia) as a virological parameter determined by quantitative PCR, and antibody responses have already been recorded and published for all pigs in this study^5^.

Seventeen Large white Babraham pigs were randomly assigned to one group of 12 and one group of five pigs. The group of 12 animals were immunized with the low virulent OUR T1988/3 isolate while the group of five were immunized with a sham vaccine. Eighteen days later all pigs were challenged with the homologous virulent OUR T1988/1 isolate of ASFV. All animals developed clinical disease. All sham and seven of the OUR T1988/3 immunized pigs reached a humane endpoint between 4 and 7 days post-infection. The last samples were collected from them on the day they were culled. Five of the OUR T1988/3 immunized pigs recovered and were eventually euthanized 35 days after the original immunization.

### Data quality

**Figure 1:**
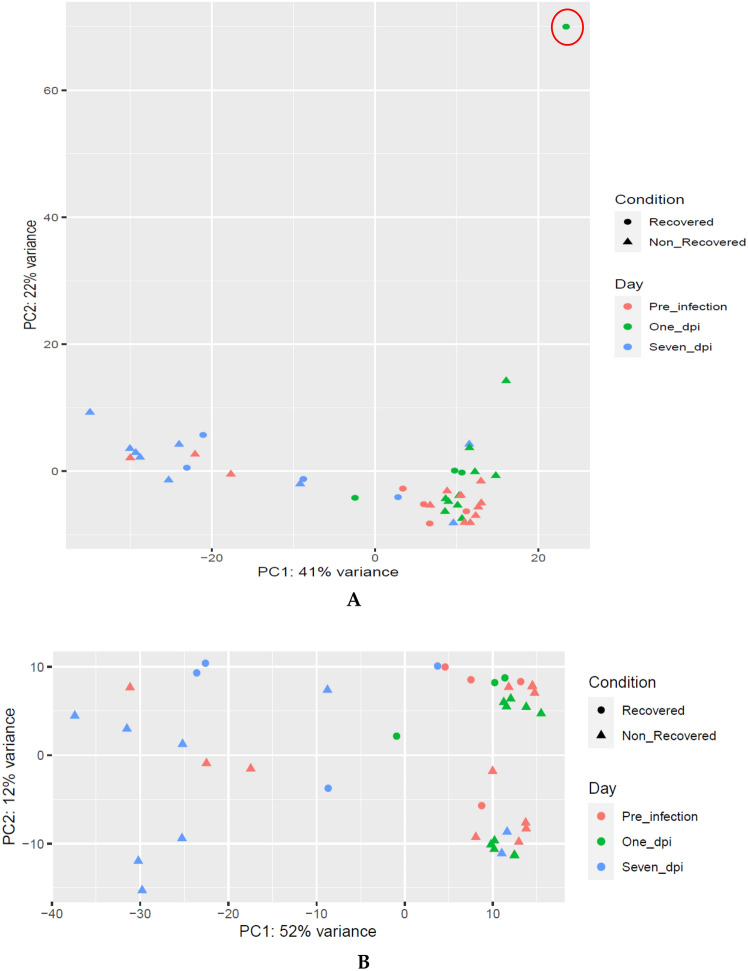
The PCA plot of all samples (**A**) and without the outlier (highlighted in red circle) sample (**B**).

Each RNA-Seq sample was individually trimmed based on its own quality control results. The number of counts pre- and post-trimming and the percentage of removed reads are shown in Supplementary Table 1. Based on the number of feature counts, the trimmed reads were normalized. Figure 1A shows that one RNA-Seq sample from one recovered pig at 1 day post-infection was an outlier (red highlighted in the related graphs) in Principle Component Analysis (PCA) and was removed in Fig. 1B. The heatmaps of the distribution of the counts for all samples (red highlighted outlier sample) and without the outlier one has been figured out in Supplementary Fig. 2A,B, respectively.

### Transcriptome assembly

Average alignment rate on the swine reference genome was 96% (the last column in Supplementary Table 1). In addition, 0.0 to 3% of reads were uniquely mapped on viral genomes. This confirms the presence of viral transcriptomes in the vaccinated pigs and is consistent with previous qPCR data showing the presence of circulating virus in these animals after challenge with OUR T1988/1^5^. As expected, the rate of mapped viral reads gradually increased for the sham-inoculated animals, reaching the highest on the day the pigs were culled (4–6 days post-infection), or 7 days after the OUR T1988/1 challenge for those that recovered (up to 3.18% of reads).

### Differential Gene Expression (DGE)

#### Non-recovered vs. recovered contrasts

**Figure 2:**
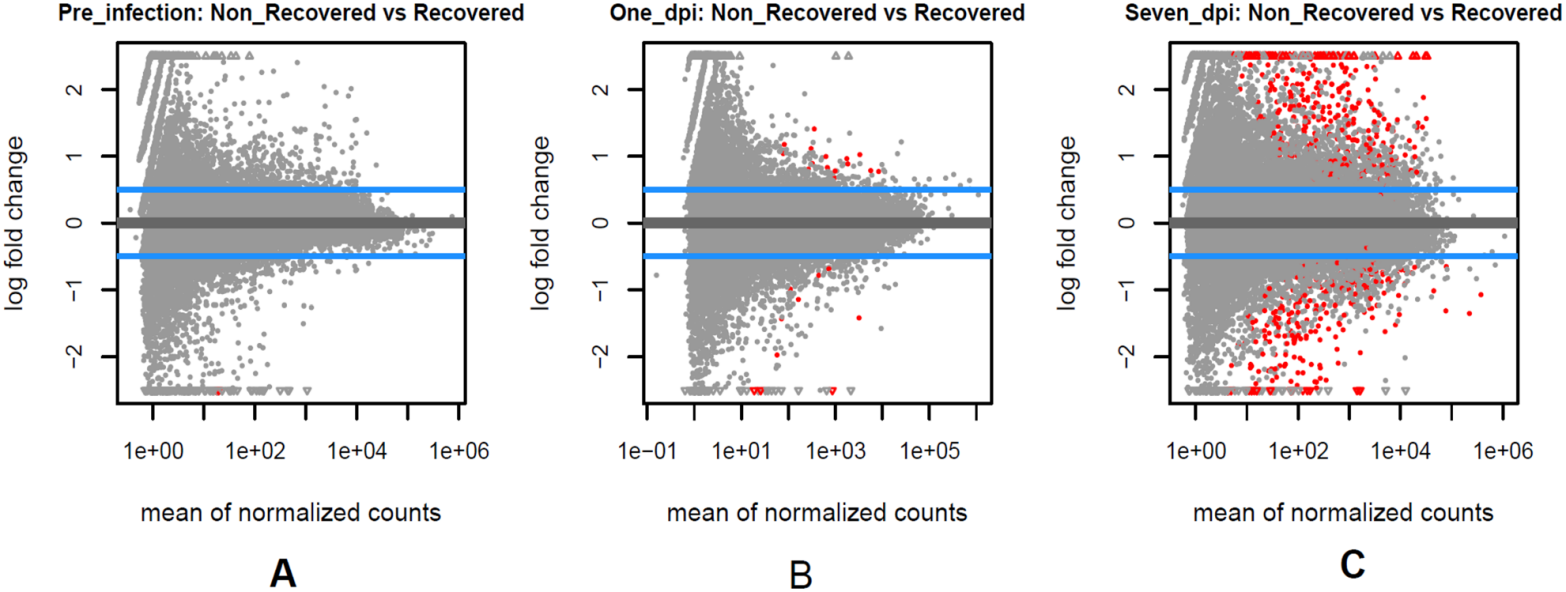
The volcano graphs of the statistically significant DEGs (Red dots) between non-recovered vs. recovered pigs (padj < 0.05) on pre-infection (**A**), 1 day (**B**), and 7 days post-infection (**C**).

**Figure 3:**
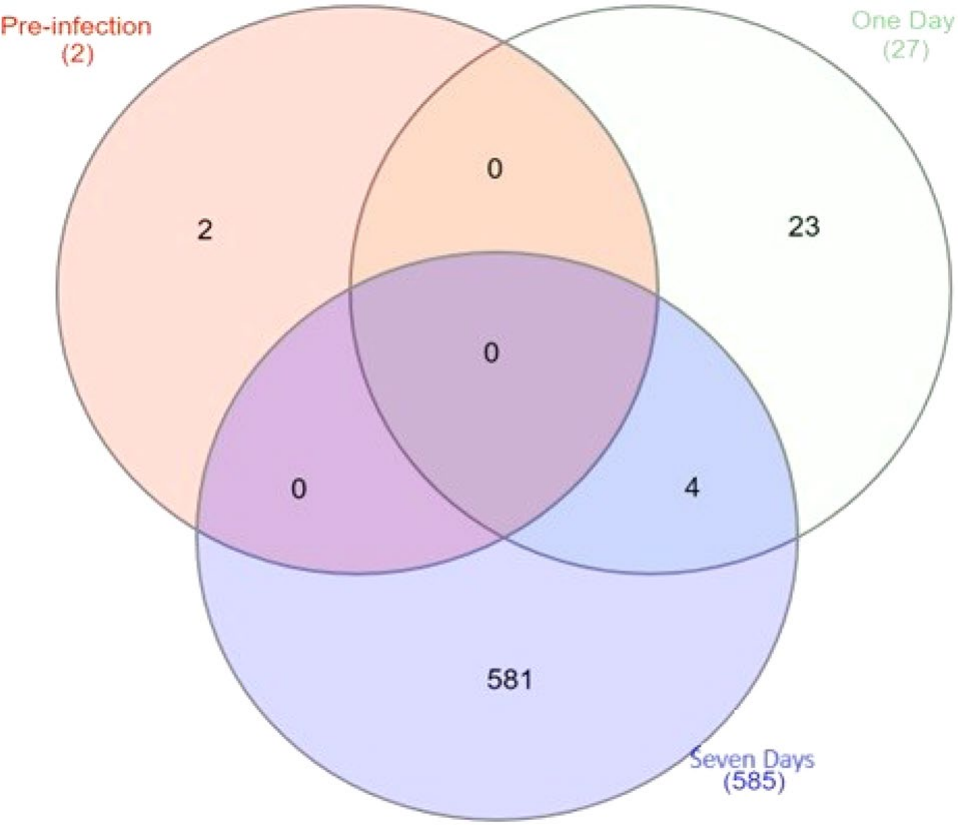
Venn diagram of the shared numbers of the statistically significant DEGs between recovered vs. non-recovered pigs (padj < 0.05) on pre-infection, 1 day, and 7 days post-infection.

The differential gene expression results of the non-recovered vs. recovered contrasts at three time points revealed 2, 37, and 558 significant differentially expressed (SDE) genes just before, 1 day, and 4–7 days post-infection (padj < 0.05), respectively (volcano graphs in Fig. 2 and listed in Supplementary Table 3). The number of shared SDE genes among these contrasts is shown in a Venn diagram (Fig. 3).

Only two genes (Fig. 2-A), High-Temperature Requirement Factor A3 (HTRA3) and Glutamine-Fructose-6-Phosphate Transaminase 2 (GFPT2), were significantly up-regulated pre-challenge (padj < 0.05) between recovered animals (n = 5) and those that became terminally ill (n = 12). The recovered pigs had a significant down-regulation of HTRA3 and GFPT2 of up to 12.85 and 9.57 fold, respectively, before the infection challenge compared to recovered pigs (Table 1). These two genes deserve some closer attention. Given that they are differentially expressed prior to the infection with the virulent strain, they could be promising biomarkers for recovery from virulent ASFV and increased resilience. HTRA3 is part of a highly conserved gene family of serine proteases^33^. GFTP2 controls the flux of glucose into the hexosamine pathway^34^.

**Table 1:** The details of the Differentially Expressed Genes (DGEs) pre-infection.

DEGs	Ensemble ID	Chromosome	Position	log2FoldChange	padj
HTRA3	ENSSSCG00000008723	8	2793797–2820185	− 3.684	0.001767
GFPT2	ENSSSCG00000014012	2	78285203–78329002	− 3.259	0.046695

By excluding naive (sham) pigs from the non-recovered group in the recovered vs. non-recovered contrast (Supplementary Table 4), two additional genes located on chromosome six, TDRD12 (Tudor Domain Containing 12) and an unknown gene (ENSSSCG00000035336), were differentially expressed (padj < 0.05). TDRD12 is essential for secondary PIWI interacting RNA biogenesis (piRNAs). piRNAs are a class of small RNAs that are used to specifically guide the DNA methylation machinery to the transposon DNA elements^32^. Interfering with host mRNA and protein synthesis is a common virulence mechanism employed by ASFV^33^.

#### Post-infection overtime contrasts among non-recovered pigs

**Figure 4:**
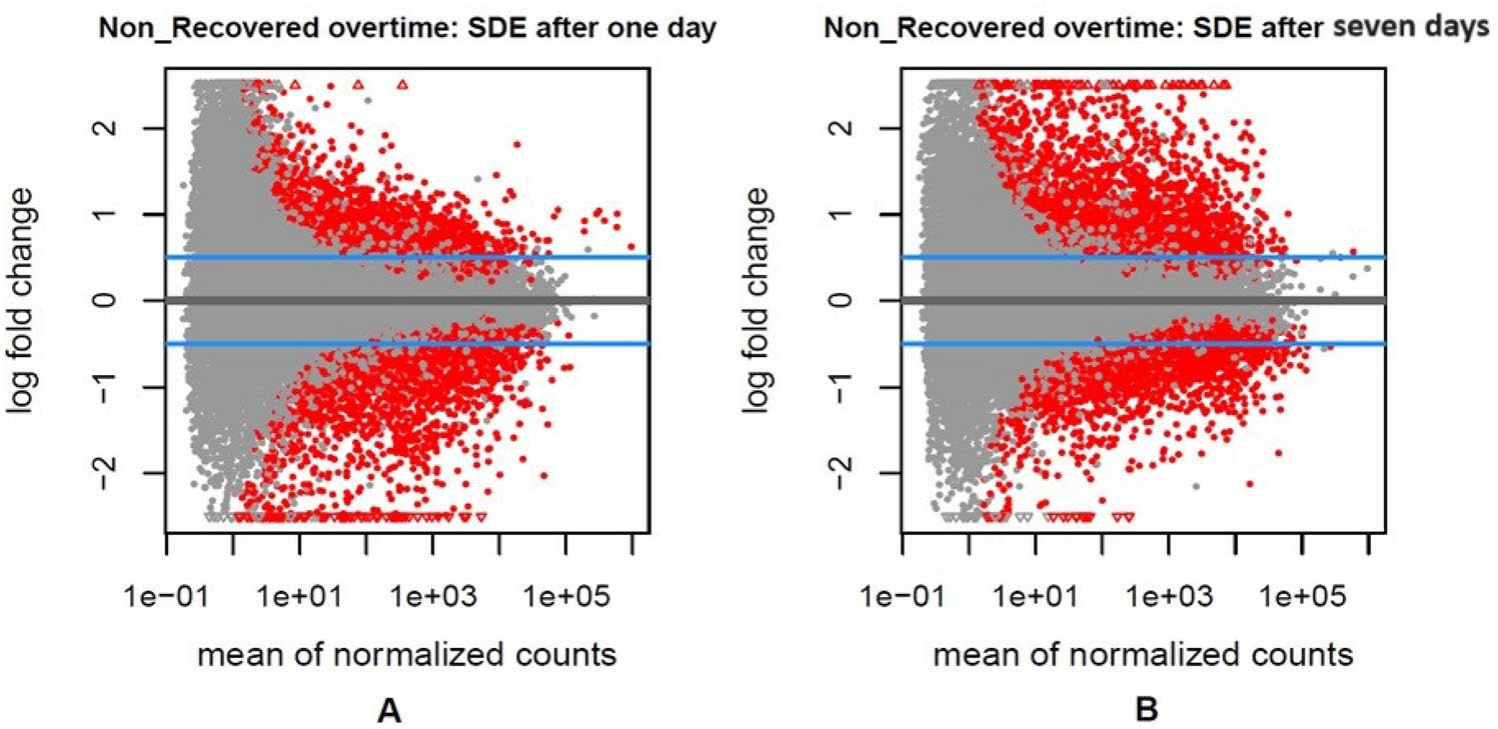
The volcano graphs of the statistically significant DEGs (Red dots) between non-recovered pigs (padj < 0.05) 1 day (**A**) and 7 days (**B**) post-infection compared with pre-infection.

**Figure 5:**
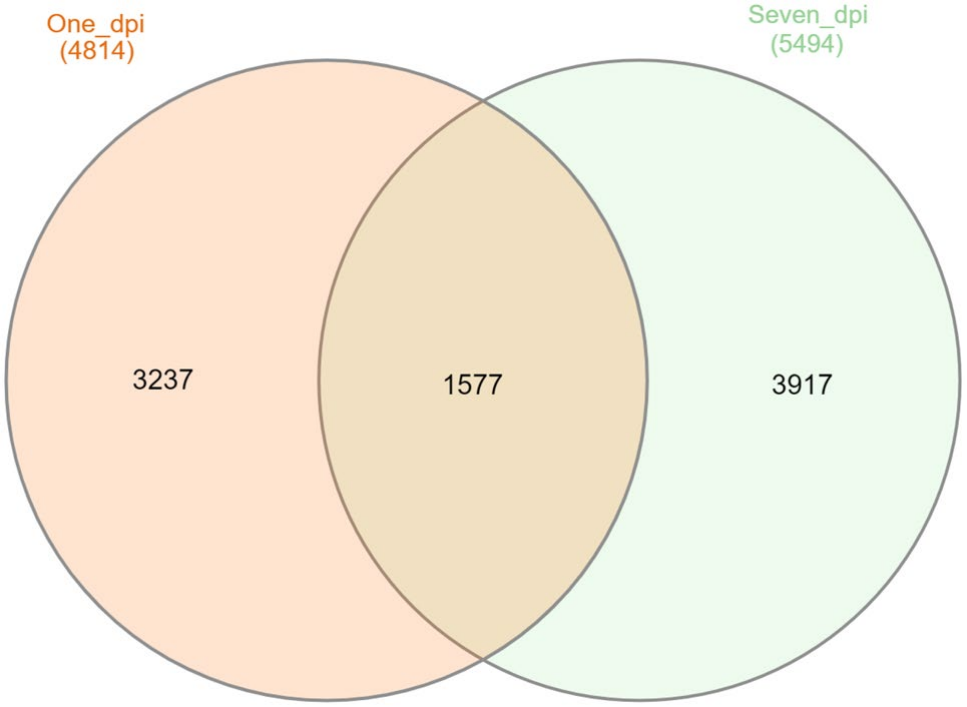
Venn diagram of the shared numbers of the statistically significant DEGs between non-recovered pigs (padj < 0.05) over time post-infection compared to pre-infection.

4814 and 5494 significant differentially genes were involved in non-recovered pigs 1 day and 7 days post-infection compared to pre-infection (padj < 0.05), respectively (Fig. 4 and Supplementary Table 5). Totally, 1577 genes were shared between 1 day and 4–7 days post-infection (Fig. 5).

#### Immunization contrasts

**Figure 6:**
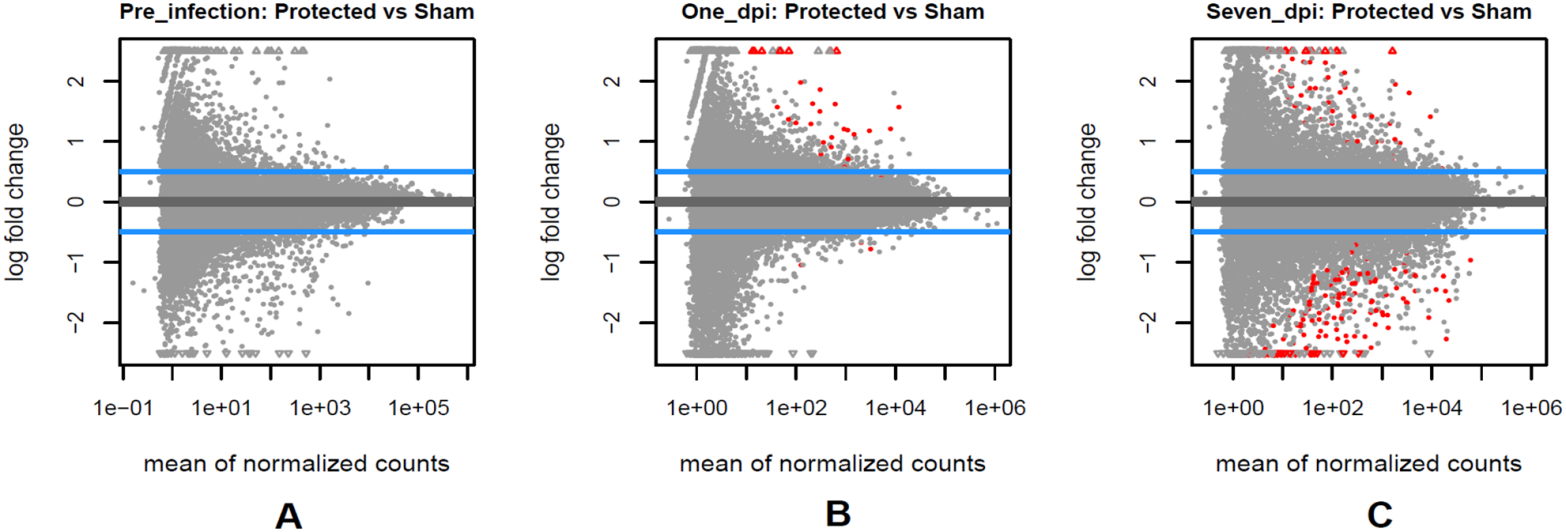
The volcano graphs of the statistically significant DEGs (Red dots) between non-protected vs. sham pigs (padj < 0.05) on pre-infection (**A**), 1 day (**B**), and 7 days post-infection (**C**).

No differentially expressed genes were significant for the contrast of vaccinated (n = 12) versus sham pigs (n = 5) in pre-infection (Fig. 6-A and Supplementary Table 6). This shows no clear gene expression signature of the immunization 18 days after the inoculations (padj < 0.05). There were 32 and 183 DEGs (padj < 0.05) in 1 day and 4–7 days post-infection between vaccinated and sham pigs (Fig. 6-B,C, and Supplementary Table 6), respectively.

The results of the immunization subgroups (Supplementary Fig. 3) were almost concordant with the correspondent recovered vs. non-recovered contrasts (Figs. 2 and 4). It re-confirms that no transcriptomic footprints resulted from the immunization 18 days before the infection challenge. Among the clinically ill pigs, only 23 and 25 genes differentiated the immunized from sham infected pigs 1 day (Supplementary Fig. 3-A2) and 4–7 days post-infection (Supplementary Fig. 3-A3), respectively. There was no overlap between the 2 days. This confirmed the previously reported cellular and humoral responses between the immunized and sham-vaccinated pigs^5^.

#### Gene Ontology (GO) and pathway enrichment analysis

There were no enriched GO terms and pathways in the recovered versus non-recovered contrasts pre-infection and 1 day after the infection challenge (Bonferroni corrected p-value < 0.05). The enriched GO terms and pathways in this contrast 7 days after the infection challenge were relevant to the immune system and summarized in Table 2.

**Table 2:** The significant enriched GO and pathways (P > 0.05) in recovered vs non-recovered contrast 7 days post-infection.

Enriched GO/pathway	Description	Gene counts	over/under	P value* < 0.05
GO biological process	Immune system process (GO:0002376)	76	+	1.05E − 02
GO cellular component	Side of membrane (GO:0098552)	32	+	8.62E − 03
Reactome pathways	Immune System (R-SSC-168256)	71	+	3.26E − 03
	Innate Immune System (R-SSC-168249)	38	+	4.41E − 03
	Toll-like Receptor Cascades (R-SSC-168898)	9	+	3.92E − 02

*Bonferroni Correction.

There were many enriched GO, KEGG pathways, and protein classes (Bonferroni corrected p-value > 0.05) over time, 1 day and 7 days post-infection compared to pre-infection. The total numbers are summarized in Table 3 and more detailed in Supplementary Materials S3. This is consistent with infection with ASFV invoking a strong inflammation reaction in the pigs.

**Table 3:** The number of the significant enriched of GO and pathways (P > 0.05) among non-recovered pigs over time. BP: Biological Process, MF: Molecular Function, CC: Cellular Components.

	GO-BP	GO-CC	GO-MF	PANTHERPathway	Reactome pathway	PANTHER protein class
One day pi	244	72	84	4	44	20
Seven days pi	197	63	65	9	51	20

### Variant calling

**Figure 7:**
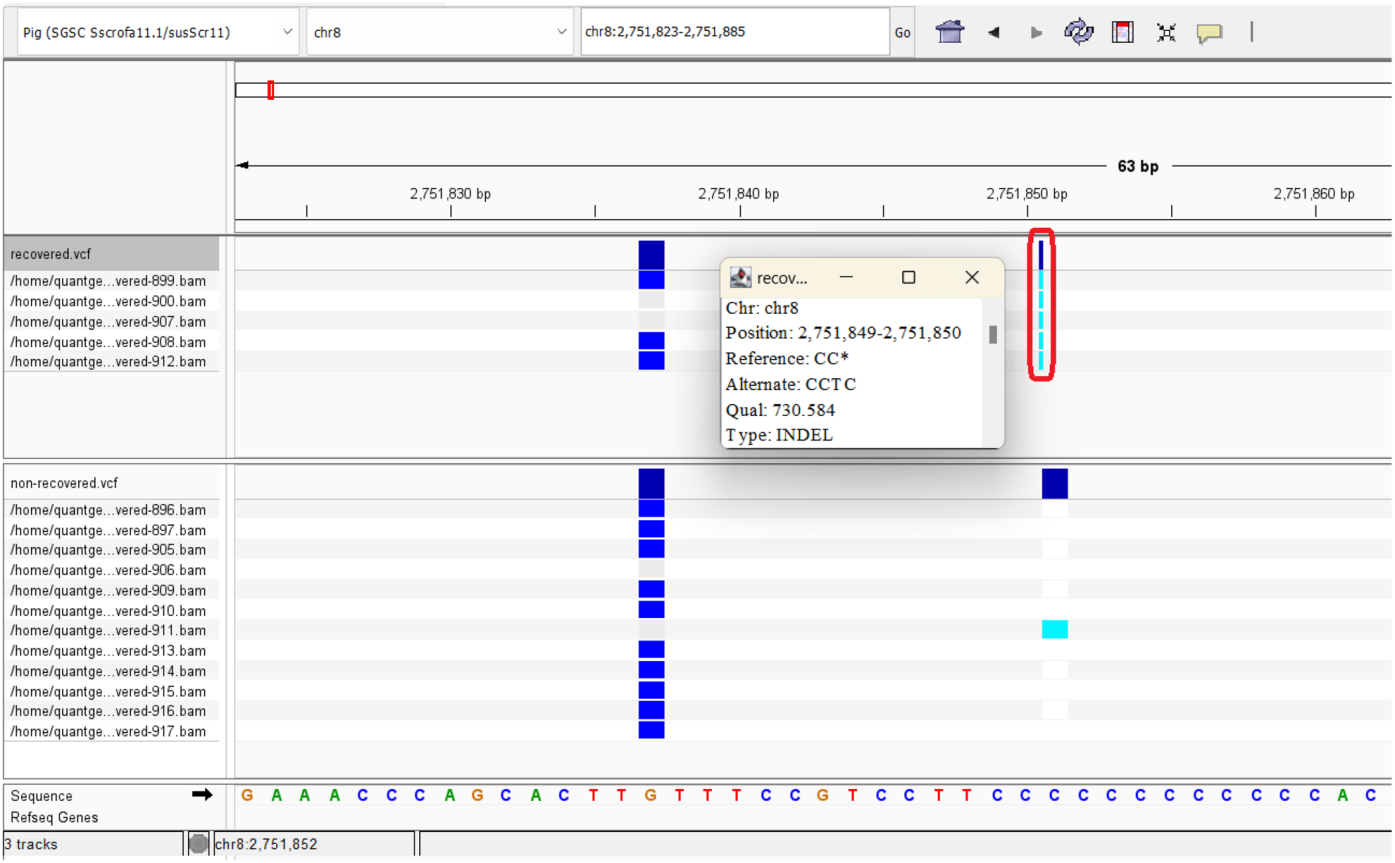
The 2-bp insertion on the sequence of the ACOX3 gene located near to HTRA3 gene (highlighted in red box) was observed only in the recovered pigs.

A total of 4,840,591 variants were discovered in the assembled transcriptome sequences of all animals, including 4,647,453 Single Nucleotide Variants (SNVs) and 193,138 Insertion-Deletions (INDELs). In addition, there were 3,607,398 and 2,429,614 SNVs and 176,637 and 141,608 INDELs for non-recovered and recovered animals, respectively, when compared separately. Upstream of HTRA3, in the sequence of ACOX3, a 2-bp insertion was detected. This InDel was shown as a homozygous insertion (CCTC/CCTC) only in the recovered pigs by IGV v.2.14.1 (Fig. 7).

## Discussion

This study aimed to use transcriptomics to identify potential candidate genes that may provide insights into differences in ASF disease outcomes. Four studies have already investigated the transcriptome profiling of the host in response to ASF. Two studies compared the pathogenesis of different strains^13,14^, one study investigated the effect of varied doses of the inoculated virus on host transcriptome profiling^15^, and one studied features of immune response of acutely infected, dead, and subclinical infection of ASFV in pigs^16^. To our knowledge this is the first report of the transcriptomic profile of pigs immunized with a low virulent isolate of ASFV before and after challenge with virulent virus. If validated, it could provide selectable biomarkers for recovery to ASF following prior immunization. In addition, a genomic variation in the ACOX3 gene sequence was observed only in the recovered pigs. It may support identifying animals that are likely to be protected because ACOX3 is closely upstream of High-Temperature Requirement Factor A3 (HTRA3), one of two highly up-regulated DEGs on pre-infection in the pigs that recovered after the challenge.

HTRA3 is hypothesized to be involved in apoptosis and is differentially expressed in a range of cancers that have been previously reviewed^33^. HTRA3 was previously shown to exhibit significantly suppressed expression of up to 5.6× under heat stress in different human cell lines^35^. HTRA3 is even more interesting because of an upstream insertion in the ACOX3 gene that putatively regulates the expression of HTRA3, where the recovered animals are homozygous for the insertion. The interaction between the two genes has already been reported in human cell lines where exposure at 42 °C for 2 h resulted in reduced expression of both ACOX3 and HTRA3^35^. It has been shown that in-vitro upregulation of HTRA3 decreased the secretion of inflammatory cytokines in artificially challenged myoblasts, while downregulation of HTRA3 led to increased secretion of cytokines^36^. This negative correlation between HTRA3 expression and secretion of inflammatory cytokines could explain the observed upregulation of HTRA3 in recovered pigs. Whether the upregulation of HTRA3 in pigs that are homozygous for the insertion was a result of the prior immunization or whether these pigs have naturally higher levels of HTRA3 requires further study. If the effect of this insertion on HTRA3 expression and recovery to ASF can be validated, then we have a very promising selectable genomic marker. The insertion can be tested in healthy pigs with a simple DNA test facilitating the genetic selection of parents with the favorable genotype.

GFTP2, the other differentially expressed gene before infection, is most likely involved in regulating the hexosamine biosynthetic pathway (HBP) and the availability of precursors for N- and O-linked glycosylation of proteins. HBP plays a key role in metabolism, health, and aging. Its rate-limiting enzyme glutamine fructose-6-phosphate amidotransferase (GFPT/GFAT) controls it. The regulation by GFPT1, GFPT2, and GFPT2:GFPT1 ratio is well studied, but other HBP regulators have remained obscure^34^. It has also been implicated in ovarian cancer, where overexpression is correlated with poor survival^37^. Since several immunological factors such as glycosylation, interferons, cells surface receptors, antibodies, etc., are contributed to GFTP2 gene function, single-cell mRNA sequencing may explain how overexpression of the GFTP2 gene would benefit the recovery of pigs.

Jaing et al. (2017) compared the host responses to two ASFV strains, including the low pathogenic OUR T1988/3 (the same strain we used as a vaccine to protect against ASF), versus the highly pathogenic Georgia 2007/1. There are some overlapping DGEs with our study. They reported the high upregulation of the host PPP1R15A gene following Georgia 2007/1 infection. This gene is a host cell homolog of the DP71L gene in ASFV. DP71L identifies a redundant mechanism to ensure that host cell translation remains intact^38^. Machuka et al.^15^ also reported the upregulation of the PPP1R15A gene during AFSV infection.

As Jaing et al. (2017) and Machuka et al.^15^ have reported, our study also showed significant upregulation for some well-known genes associated with macrophages only 1 day post-infection of ASFV, including SIGLEC1, CD163, HMOX1, and S100A8. In addition, several chemokines and chemokine receptor genes, previously described as DGE during acute ASFV infection^39,40^, were significantly differentially expressed. It has been suggested that the upregulation of the macrophage surface marker gene, SIGLEC1, relates to increased circulating monocytes. CD163, a second macrophage-associated marker gene, further supports increased macrophages. The increased numbers of circulating CD163-positive monocytes during acute ASFV infection have been reported^41^. CD163 has been proposed as a candidate receptor for ASFV. An in vitro experiment indicated that the PRRSV receptor CD163 may be playing a role in ASFV infectivity. ASFV-infected macrophages had an enhanced expression of CD163, and anti-CD163 antibodies could block infection of ASFV in macrophages in a dose-dependent manner^42^. However, the challenge of CD163-knockout pigs with the Georgia 2007/1 isolate of ASFV resulted in clinical signs consistent with acute disease and ruled out a significant role for CD163 in infection^43^. As it has already been reported^13^, the upregulation of HMOX1 was observed to be linked with increased CD163 expression. HMOX1 codes a protein found in CD163-positive macrophages. Single-cell sequencing on macrophages may result in high-resolution conclusions.

The RELA protein, a subunit of the NF-KB transcription factor, plays a key role in regulating immune response upon infection. A three amino acid difference in the RELA protein between the warthog and domestic pigs was used as a base for gene editing of domestic pigs. Pigs with three warthog amino acid substitutions had a delayed onset of clinical signs and less viral DNA in blood and nasal samples after challenge with ASFV. Functional studies have revealed that the polymorphic sequence variation S531P in RELA proto-oncogene, as a gene associated with resistance/tolerance to ASF, promotes most of the distinct host response to ASFV in warthogs and domestic pigs^44^. The edited porcine RELA gene was then delivered to the pig zygote, resulting in the live born of edited piglets^45^. In our study, RELA was downregulated only 7 days post-infection. Machuka et al.^15^ reported the down-regulation of RELA in the high- and medium-dose groups of ASFV but not differentially expressed in the low dose group. It can be concluded the gene modifications of RELA^43,45–47^ and CD163^43^ genes have not been successful for ASFV resistance compared with gene editing for PRRSV^48^ or TGEV^49^. Two suggested DEGs can be new potential targets to gene editing for resistance to ASF.

We found some differentially expressed miRNA during ASFV infection, including ssc-mir-23a, ssc-mir-29c, ssc-mir-186, and miR-1296 that have been previously reported between pigs infected with attenuated and virulent ASFV isolates^50^. Another differentially expressed miRNA, MIR142 was differentially expressed between infected and non-infected pigs in a previous study^51^. As additional differentially expressed miRNA: ssc-mir-27a, ssc-mir-1271, ssc-mir-24-1, ssc-mir-7-2, ssc-mir-6782, ssc-mir-9841, ssc-mir-10390, ssc-mir-425, ssc-mir-29b-2, ssc-mir-503, and MIR335 differed from those reported by previous studies^13,50,51^.

Interferons are a key factor of cellular immune response in protecting pigs from ASF. The secretion of IFNγ by lymphocytes correlates with the degree of protection of animals immunized with low-virulent isolates from infection by a homologous virulent strain^20,52–54^. There was the same condition in the present study. The IFNγ gene showed significant differential expression only 7 days post-infection.

It has already been shown that the African Swine Fever virus induces STAT1 and STAT2 degradation to counteract IFN-I signaling^55^. The upregulation of STAT1 and STAT2 genes in the present study may confirm the mentioned mechanism.

The day after challenge shows only a limited gene expression signature between recovered and non-recovered animals, while the gene expression differences 7 days post-infection mainly indicate expected differences between recovering animals and those succumbing to ASF. Also, it has already showed the numbers of CD4+ CD8−, CD3+ CD8−, and CD4+ CD8− cells were elevated in the immunized pigs 18 and 19 days after immunization compared to the shams (control group)^5^. With this hindsight, it would have been desirable to have samples from earlier time points before, and as, the animals began to show clinical signs (2, 3 and 4 days post-infection), rather than once the animals had developed acute disease.

## Conclusion

The current study could present a few promising transcriptomic and one potential genomic biomarker to distinguish differential susceptibility in response to ASFV challenge and also vaccination outcomes with some overlapping. These preliminary results need to be tested in more trials, with more sampling points after the challenge. It would also be important to test these differences in outbred farm pigs.

### Supplementary Information

Supplementary Information 1.

Supplementary Information 2.

Supplementary Information 3.

Supplementary Table S1.

Supplementary Table S2.

Supplementary Table S3.

Supplementary Table S4.

Supplementary Table S5.

Supplementary Table S6.

Supplementary Figure S1.

Supplementary Figure S2.

Supplementary Figure S3.

## Data Availability

Raw RNA-Seq reads are publicly available on the EBI ArrayExpress depository (https://www.ebi.ac.uk/biostudies/arrayexpress/) with accession number E-MTAB-12608.

## References

[1] Salguero FJ (2020). Comparative pathology and pathogenesis of African swine fever infection in swine. Front. Vet. Sci..

[2] Cwynar P, Stojkov J, Wlazlak K (2019). African swine fever status in Europe. Viruses.

[3] Pikalo J, Zani L, Hühr J, Beer M, Blome S (2019). Pathogenesis of African swine fever in domestic pigs and European wild boar—Lessons learned from recent animal trials. Virus Res..

[4] (WOAH), W. O. f. A. H. 35th Situation Report on African Swine Fever (ASF). (2023).

[5] Goatley LC (2022). Cellular and humoral immune responses after immunisation with low virulent african swine fever virus in the large white inbred Babraham line and outbred domestic pigs. Viruses..

[6] Cackett G (2020). The African swine fever virus transcriptome. J. Virol..

[7] Cackett G, Sýkora M, Werner F (2020). Transcriptome view of a killer: African swine fever virus. Biochem. Soc. Trans..

[8] Torma G (2021). Combined short and long-read sequencing reveals a complex transcriptomic architecture of African Swine Fever Virus. Viruses..

[9] Pérez-Sánchez, R., Carnero-Morán, Á., Soriano, B., Lloréns, C. & Oleaga, A. RNA-seq analysis and gene expression dynamics in the salivary glands of the argasid tick Ornithodoros erraticus along the trophogonic cycle. *Parasites Vectors*. **14**, 170. 10.1186/s13071-021-04671-z (2021). 10.1186/s13071-021-04671-z PMC7980729 33743776

[10] Ju X (2021). Genome-wide transcriptomic analysis of highly virulent African swine fever virus infection reveals complex and unique virus host interaction. Vet. Microbiol..

[11] Cackett G, Portugal R, Matelska D, Dixon L, Werner F (2022). African swine fever virus and host response: Transcriptome profiling of the Georgia 2007/1 strain and porcine macrophages. J. Virol..

[12] Zheng Y (2022). Transcriptome profiling in swine macrophages infected with African swine fever virus at single-cell resolution. Proc. Natl. Acad. Sci..

[13] Jaing C (2017). Gene expression analysis of whole blood RNA from pigs infected with low and high pathogenic African swine fever viruses. Sci. Rep..

[14] Kholod N, Koltsov A, Koltsova G (2022). Analysis of gene expression in monocytes of immunized pigs after infection with homologous or heterologous African swine fever virus. Front. Vet. Sci..

[15] Machuka, E. M.* et al.* (2022) Transcriptome profile of spleen tissues from locally-adapted Kenyan pigs (Sus scrofa) experimentally infected with three varying doses of a highly virulent African swine fever virus genotype IX isolate: Ken12/busia.1 (ken-1033). *BMC Genom*. **23**, 522. 10.1186/s12864-022-08754-8. 10.1186/s12864-022-08754-8 PMC9294756 35854219

[16] Sun H (2021). Transcriptome profiling reveals features of immune response and metabolism of acutely infected, dead and asymptomatic infection of African Swine Fever virus in pigs. Front. Immunol..

[17] Netherton CL (2019). Identification and immunogenicity of African Swine Fever Virus Antigens. Front. Immunol..

[18] Schwartz JC (2018). The major histocompatibility complex homozygous inbred Babraham pig as a resource for veterinary and translational medicine. HLA.

[19] Galindo-Cardiel I (2013). Standardization of pathological investigations in the framework of experimental ASFV infections. Virus Res..

[20] King K (2011). Protection of European domestic pigs from virulent African isolates of African swine fever virus by experimental immunisation. Vaccine.

[21] King DP (2003). Development of a TaqMan PCR assay with internal amplification control for the detection of African swine fever virus. J. Virol. Methods.

[22] Bolger AM, Lohse M, Usadel B (2014). Trimmomatic: A flexible trimmer for Illumina sequence data. Bioinformatics.

[23] Bushnell, B. in *9th Annual Genomics of Energy & Environment Meeting.*

[24] Kim D, Paggi JM, Park C, Bennett C, Salzberg SL (2019). Graph-based genome alignment and genotyping with HISAT2 and HISAT-genotype. Nat. Biotechnol..

[25] Chapman DAG, Tcherepanov V, Upton C, Dixon LK (2008). Comparison of the genome sequences of non-pathogenic and pathogenic African swine fever virus isolates. J. General Virol..

[26] de Villiers EP (2010). Phylogenomic analysis of 11 complete African swine fever virus genome sequences. Virology.

[27] Liao Y, Smyth GK, Shi W (2013). featureCounts: An efficient general purpose program for assigning sequence reads to genomic features. Bioinformatics.

[28] Love MI, Huber W, Anders S (2014). Moderated estimation of fold change and dispersion for RNA-seq data with DESeq2. Genome Biol..

[29] R: A language and environment for statistical computing (R Foundation for Statistical Computing, Vienna, Austria, 2022).

[30] Thomas PD (2022). PANTHER: Making genome-scale phylogenetics accessible to all. Protein Sci..

[31] Danecek P (2021). Twelve years of SAMtools and BCFtools. Gigascience..

[32] Robinson JT, Thorvaldsdóttir H, Wenger AM, Zehir A, Mesirov JP (2017). Variant review with the integrative genomics viewer. Cancer Res..

[33] Wang Y, Nie G (2021). Overview of human HtrA family proteases and their distinctive physiological roles and unique involvement in diseases, especially cancer and pregnancy complications. Int. J. Mol. Sci..

[34] Kroef V (2022). GFPT2/GFAT2 and AMDHD2 act in tandem to control the hexosamine pathway. eLife.

[35] Feliciello I, Sermek A, Pezer Ž, Matulić M, Ugarković Đ (2020). Heat stress affects H3K9me3 level at human alpha satellite DNA repeats. Genes.

[36] Shen Z (2022). High temperature requirement A3 attenuates hypoxia/reoxygenation induced injury in H9C2 cells via suppressing inflammatory responses. Eur. J. Pharmacol..

[37] Zhou L (2019). Glutamine-fructose-6-phosphate transaminase 2 (GFPT2) promotes the EMT of serous ovarian cancer by activating the hexosamine biosynthetic pathway to increase the nuclear location of β-catenin. Pathol. Res. Practice.

[38] Zhang F, Moon A, Childs K, Goodbourn S, Dixon LK (2010). The African Swine Fever Virus DP71L protein recruits the protein phosphatase 1 catalytic subunit to dephosphorylate eIF2α and inhibits chop induction but is dispensable for these activities during virus infection. J. Virol..

[39] Fishbourne E, Abrams CC, Takamatsu H-H, Dixon LK (2013). Modulation of chemokine and chemokine receptor expression following infection of porcine macrophages with African swine fever virus. Vet. Microbiol..

[40] Fishbourne E (2013). Increase in chemokines CXCL10 and CCL2 in blood from pigs infected with high compared to low virulence African swine fever virus isolates. Vet. Res..

[41] Karalyan Z (2012). Pathology of porcine peripheral white blood cells during infection with African swine fever virus. BMC Vet. Res..

[42] Sánchez-Torres C (2003). Expression of porcine CD163 on monocytes/macrophages correlates with permissiveness to African swine fever infection. Arch. Virol..

[43] Popescu L (2017). Genetically edited pigs lacking CD163 show no resistance following infection with the African swine fever virus isolate, Georgia 2007/1. Virology.

[44] Palgrave CJ (2011). Species-specific variation in RELA underlies differences in NF-κB activity: A potential role in African swine fever pathogenesis. J. Virol..

[45] Lillico SG (2013). Live pigs produced from genome edited zygotes. Sci. Rep..

[46] Lillico SG (2016). Mammalian interspecies substitution of immune modulatory alleles by genome editing. Sci. Rep..

[47] McCleary S (2020). Substitution of warthog NF-κB motifs into RELA of domestic pigs is not sufficient to confer resilience to African swine fever virus. Sci. Rep..

[48] Tu C-F, Chuang C-K, Yang T-S (2022). The application of new breeding technology based on gene editing in pig industry—A review. Anim. Biosci..

[49] Xu K (2020). CD163 and pAPN double-knockout pigs are resistant to PRRSV and TGEV and exhibit decreased susceptibility to PDCoV while maintaining normal production performance. eLife.

[50] Núñez-Hernández F (2017). Differential expression of porcine microRNAs in African swine fever virus infected pigs: A proof-of-concept study. Virol. J..

[51] Pang Z (2023). Identification of potential miRNA–mRNA regulatory network associated with regulating immunity and metabolism in pigs induced by ASFV infection. Animals.

[52] Monteagudo PL (2017). BA71ΔCD2: A new recombinant live attenuated African Swine Fever Virus with cross-protective capabilities. J. Virol..

[53] Takamatsu HH (2013). Cellular immunity in ASFV responses. Virus Res..

[54] Lacasta A (2015). Live attenuated African swine fever viruses as ideal tools to dissect the mechanisms involved in viral pathogenesis and immune protection. Vet. Res..

[55] Riera E (2021). African Swine Fever Virus induces STAT1 and STAT2 degradation to counteract IFN-I signaling. Front. Microbiol..

