# Supplementary figures and images for: The transcriptomic insight into the differential susceptibility of African Swine Fever in inbred pigs
Source: Sci Rep. 2024 Mar 11;14:5944. doi: 10.1038/s41598-024-56569-2 (PMC10928096; doi:10.1038/s41598-024-56569-2)

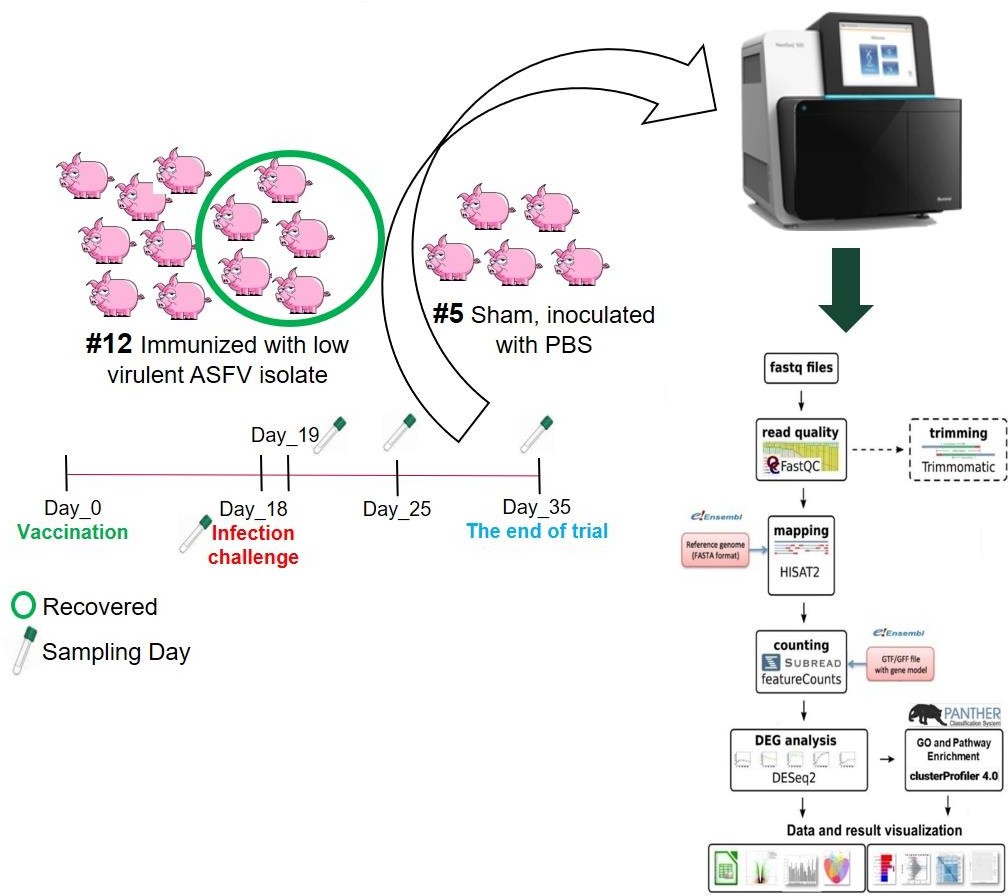


Supplementary Figure 1 (FigS1): The schematic experiment design and computational workflow.

Supplement: Supplementary file 10 — Supplementary Figure S1. [file 41598_2024_56569_MOESM10_ESM.docx]
