# Supplementary material for: The transcriptomic insight into the differential susceptibility of African Swine Fever in inbred pigs
Source: Sci Rep. 2024 Mar 11;14:5944. doi: 10.1038/s41598-024-56569-2 (PMC10928096; doi:10.1038/s41598-024-56569-2)
Supplement: Supplementary file 11 — Supplementary Figure S2. [file 41598_2024_56569_MOESM11_ESM.docx]

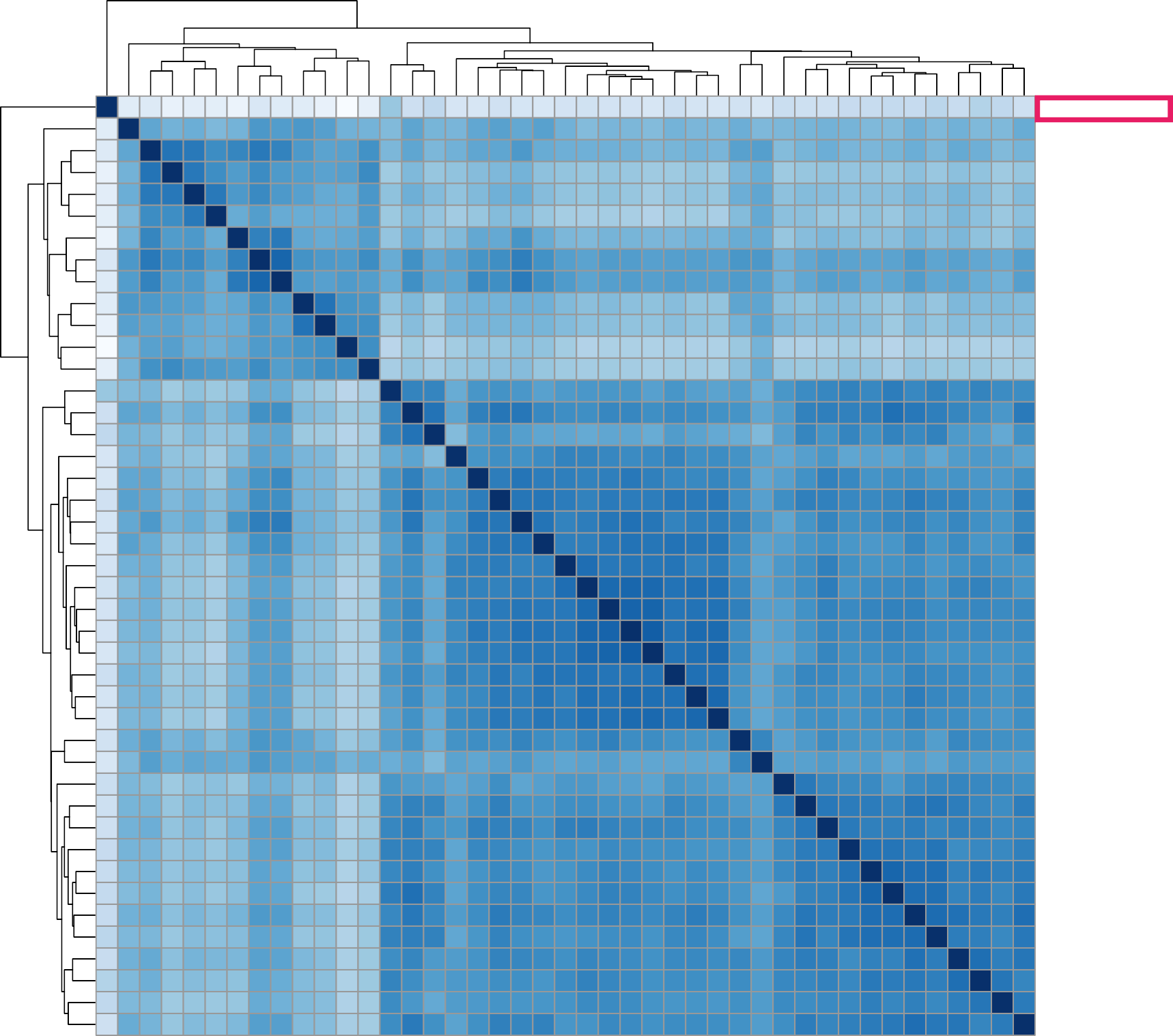
912_Recovered_One_dpi 907_Recovered_Seven_dpi 909_Non_Recovered_Seven_dpi 915_Non_Recovered_Seven_dpi 905_Non_Recovered_Seven_dpi 906_Non_Recovered_Seven_dpi 917_Non_Recovered_Seven_dpi 905_Non_Recovered_Pre_infection 915_Non_Recovered_Pre_infection 900_Recovered_Seven_dpi 912_Recovered_Seven_dpi 896_Non_Recovered_Pre_infection 897_Non_Recovered_Seven_dpi 911_Non_Recovered_One_dpi 897_Non_Recovered_Pre_infection 914_Non_Recovered_Seven_dpi 908_Recovered_Pre_infection 900_Recovered_Pre_infection 917_Non_Recovered_Pre_infection 908_Recovered_One_dpi 912_Recovered_Pre_infection 916_Non_Recovered_Pre_infection 909_Non_Recovered_Pre_infection 899_Recovered_Pre_infection 911_Non_Recovered_Pre_infection 913_Non_Recovered_Pre_infection 910_Non_Recovered_Pre_infection 906_Non_Recovered_Pre_infection 914_Non_Recovered_Pre_infection 899_Recovered_Seven_dpi 913_Non_Recovered_Seven_dpi 917_Non_Recovered_One_dpi 905_Non_Recovered_One_dpi 913_Non_Recovered_One_dpi 899_Recovered_One_dpi 897_Non_Recovered_One_dpi 909_Non_Recovered_One_dpi 896_Non_Recovered_One_dpi 910_Non_Recovered_One_dpi 914_Non_Recovered_One_dpi 916_Non_Recovered_One_dpi 900_Recovered_One_dpi 911_Non_Recovered_Seven_dpi

# A

140


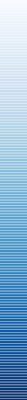
120

100

80

60

40

20

0


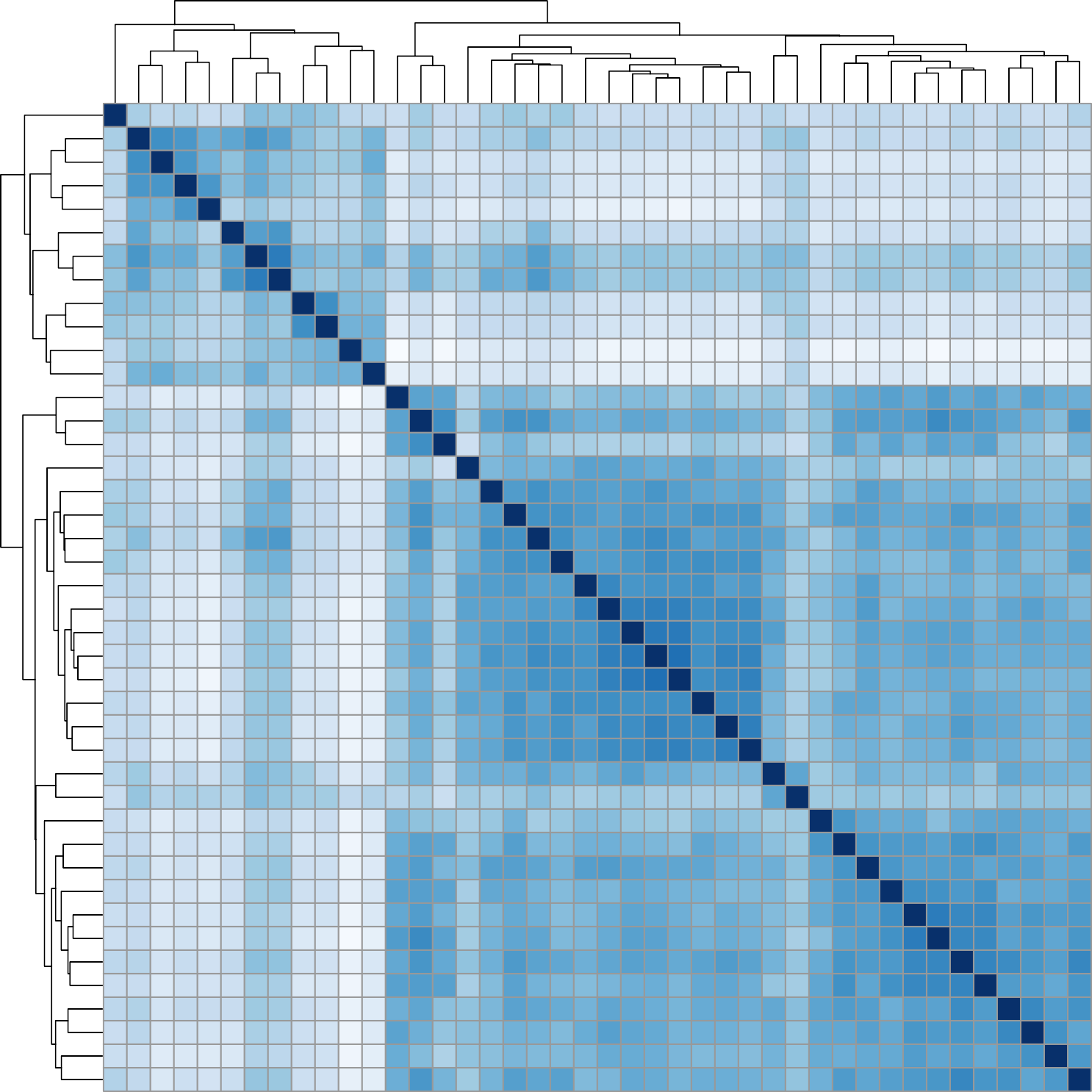
907−Recovered 909−Non_Recovered 915−Non_Recovered 905−Non_Recovered 906−Non_Recovered 917−Non_Recovered 905−Non_Recovered 915−Non_Recovered 900−Recovered 912−Recovered 896−Non_Recovered 897−Non_Recovered 911−Non_Recovered 897−Non_Recovered 914−Non_Recovered 908−Recovered 900−Recovered 917−Non_Recovered 908−Recovered 912−Recovered 916−Non_Recovered 909−Non_Recovered 899−Recovered 911−Non_Recovered 913−Non_Recovered 910−Non_Recovered 906−Non_Recovered 914−Non_Recovered 899−Recovered 913−Non_Recovered 917−Non_Recovered 905−Non_Recovered 913−Non_Recovered 899−Recovered 897−Non_Recovered 909−Non_Recovered 896−Non_Recovered 910−Non_Recovered 914−Non_Recovered 916−Non_Recovered 900−Recovered 911−Non_Recovered

# B

100


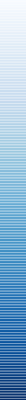
80

60

40

20

0

**Supplementary Figure 2 (FigS2): The heatmap of all samples with (A) and without the outlier sample (B), generated with the pheatmap package (version 1.0.12) in R**, <https://cran.r-project.org/web/packages/pheatmap/index.html>.
