# Supplementary material for: The transcriptomic insight into the differential susceptibility of African Swine Fever in inbred pigs
Source: Sci Rep. 2024 Mar 11;14:5944. doi: 10.1038/s41598-024-56569-2 (PMC10928096; doi:10.1038/s41598-024-56569-2)
Supplement: Supplementary file 12 — Supplementary Figure S3. [file 41598_2024_56569_MOESM12_ESM.docx]

**
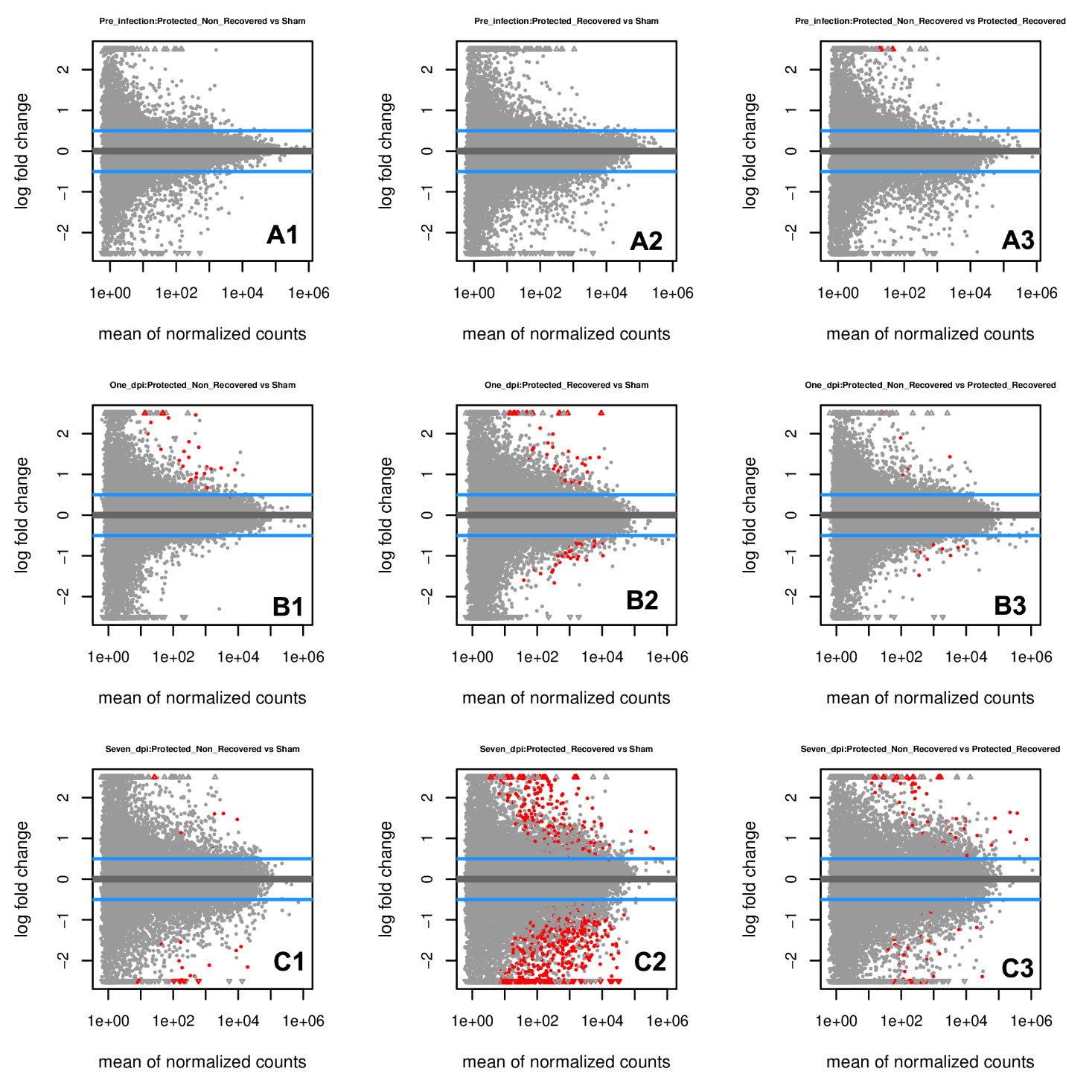
**

**Supplementary Figure 3 (FigS3): The volcano graphs of the statistically significant DEGs (Red dots) between protection subgroup pairwise contrasts including protection and recovery combinations (1-3) with sampling time (A-C) (padj < 0.05)**
